# Comparison of the fluorescein angiography-guided and indocyanine green angiography-guided photodynamic therapy in the treatment of non-resolving central serous chorioretinopathy

**DOI:** 10.1038/s41598-023-28890-9

**Published:** 2023-01-30

**Authors:** Nazanin Ebrahimiadib, Arash Mirzaei, Shaghayegh Esfandiarifard, Sonal Tuli, Ehsan Najibzadeh, Marjan Imani Fooladi, Kaveh Fadakar

**Affiliations:** 1 Department of ophthalmology, Eye Research Center, Tehran University of Medical Sciences, Tehran, Iran; 2 Department of Ophthalmology, University of Florida, Gainesville, FL USA; 3 Department of Ophthalmology, University of Pittsburgh, Pittsburgh, PA USA

**Keywords:** Medical research, Diseases, Eye diseases

## Abstract

To compare the functional and anatomical outcome of fluorescein angiography (FA) versus indocyanine green angiography (ICGA) guided photodynamic therapy (PDT) in the treatment of non-resolving central serous chorioretinopathy (CSCR). In this prospective interventional case series, all patients with non-resolving CSCR, defined as persistent SRF involving subfoveal area for at least three months, were nonrandomly assigned to receive either FA or ICGA-guided half dose PDT. Baseline and 4 months post-treatment data including best corrected visual acuity (BCVA), the status of foveal subretinal fluid, subfoveal choroidal thickness, choroidal vascularity index, pigment epithelial detachment area, treatment and PDT spot numbers were collected. Thirty-six eyes were included; 24 received ICGA-guided and 12 received FA-guided PDT. Overall, improvement in BCVA and choroidal parameters were observed in all patients. There was no significant difference in baseline parameters as well as follow-up measurements between groups. However, the mean total energy dose and spot number in the IGCA-guided PDT were significantly higher than the FA-guided PDT group (*P* = 0.001). Both FA-guided and ICGA-guided half-dose PDT were effective in the treatment of non-resolving CSCR, with favorable functional and anatomical outcome. In FA group, PDT with smaller spot sizes and fewer numbers of spots were applied.

## Introduction

Central serous chorioretinopathy (CSCR) is a retinal disorder that commonly affects men of 30 to 50-year-old. Neurosensory retinal detachment is the predominant morphological feature of this disorder, which mainly involves the macular region. Decreased visual acuity, vision distortion, relative scotoma, dyschromatopsia, micropsia, and reduced contrast sensitivity are among the early symptoms of this disorder^1^.

Most cases resolve spontaneously, however, chronic or non-resolving CSCR may progress to the atrophy of the retinal pigment epithelium (RPE) and retina with eventual vision loss^2^. Therefore, the current trend is more toward treatment rather than observation. Although several medical and laser treatments have been evaluated, photodynamic therapy (PDT) has been found to be the most effective method of treatment for acute and chronic CSCR^3–7^.

During PDT, a light-sensitive pigment called Verteporfin is employed to reduce the permeability of the choroidal arteries and therefore resolution of the subretinal fluid (SRF)^8^. Recognition of the areas of hyperpermeability of choroidal vessels, even in subclinical phase of CSCR, is best done using Indocyanine Green Angiography (ICGA)^7^. Many specialists apply PDT spots on the pathologic regions illustrated in ICGA, however, this dye is expensive and may not be available in all centers.

Fluorescein angiography (FA) can show areas of the leakage through RPE defect and is a less expensive and more accessible image modality. Koytak and colleagues applied PDT on pathologic areas shown on FA and obtained good results in terms of vision improvement and resolution of SRF^9^.

Few studies have compared FA based PDT versus ICGA based PDT for treatment of non-resolving CSCR, and the results regarding resolution of subretinal fluid, improvement in best corrected visual acuity (BCVA), central macular thickness and subfoveal choroidal thickness (SFCT) are contradictory^9–11^. In the current study, we aimed to compare changes in parameters such as BCVA, choroidal thickness, the status of SRF, choroidal vascularity index (CVI), pigment epithelial detachment (PED) area, number of applied laser spots and total PDT laser energy, between FA and ICGA-guided half-dose PDT in patients with non-resolving CSCR.

## Materials and methods

### Study population

In this prospective interventional case series, all patients with non-resolving CSCR who were referred to Farabi Eye Hospital from December 2020 to April 2021 were enrolled. Non-resolving CSCR was defined as persistent subretinal fluid involving foveal area for at least three months based on enhanced depth imaging optical coherence tomography (EDI-OCT) images.

History of steroid usage (either systemic or ocular), Cushing's syndrome, diabetic retinopathy, high myopia (6 D and above), previous PDT or focal laser photocoagulation, treatment with anti-VEGF agents, choroidal polypoid vasculopathy, congenital macular or retinal disease, pregnancy, and posterior uveitis were noted as exclusion criteria. Written informed consent letter was obtained from all participants. The ethics committee of Tehran University of Medical Sciences approved this study (Code: IR.TUMS.FARABIH.REC.1399.025). The study adhered to the tenets of the Helsinki Declaration.

Demographic data was collected and a comprehensive ocular examination and dye-based angiography (Heidelberg Spectralis, Heidelberg Engineering, Germany) was performed for all patients. Additionally, patients underwent EDI-OCT (Heidelberg Engineering Inc., Heidelberg, Germany) and OCT angiography (OCTA) (AngioVue, Optovue, Inc., Fremont, CA, USA) at baseline. Patients were non-randomly allocated to receive either FA guided or ICGA guided PDT. As our allocation was not random, there was a concern for unequal distribution of FA leakage patterns among the two groups. Therefore, from both groups, we selected eyes having both FA and ICG available, and compared the patterns of FA leakage between them. These FA leakage patterns were classified into discrete, multiple discrete, and diffuse leakage. We also evaluated areas of abnormal leakage in both FA and ICGA images. We determined whether these areas are compatible with each other, in both treated and asymptomatic fellow eye.

In FA group, areas of active leakage and in ICGA group, areas of choroidal vascular hyperpermeability in middle and late phases were considered for laser application. Meticulous attention was paid to exclude areas of window defect and staining in FA group, from laser treatment. All patients received intravenous infusion of Verteporfin (3 mg/m^2^) over eight minutes and were treated with laser two minutes later. The standard dose of 50 J/cm^2^ laser was applied for 83 s. For those who underwent ICGA, an interval of at least three days from ICGA, was considered for PDT application.

Patients were re-examined four months after PDT when thorough ophthalmic examinations, EDI-OCT and OCTA were performed. In addition, the following data was collected at baseline and four months after half-dose PDT; the treated eye, BCVA, the status of foveal subretinal fluid (FSRF), SFCT, CVI, PED area, total PDT laser energy dose, area and number of spots applied.

### Image acquisition and analysis protocol

**Figure 1:**
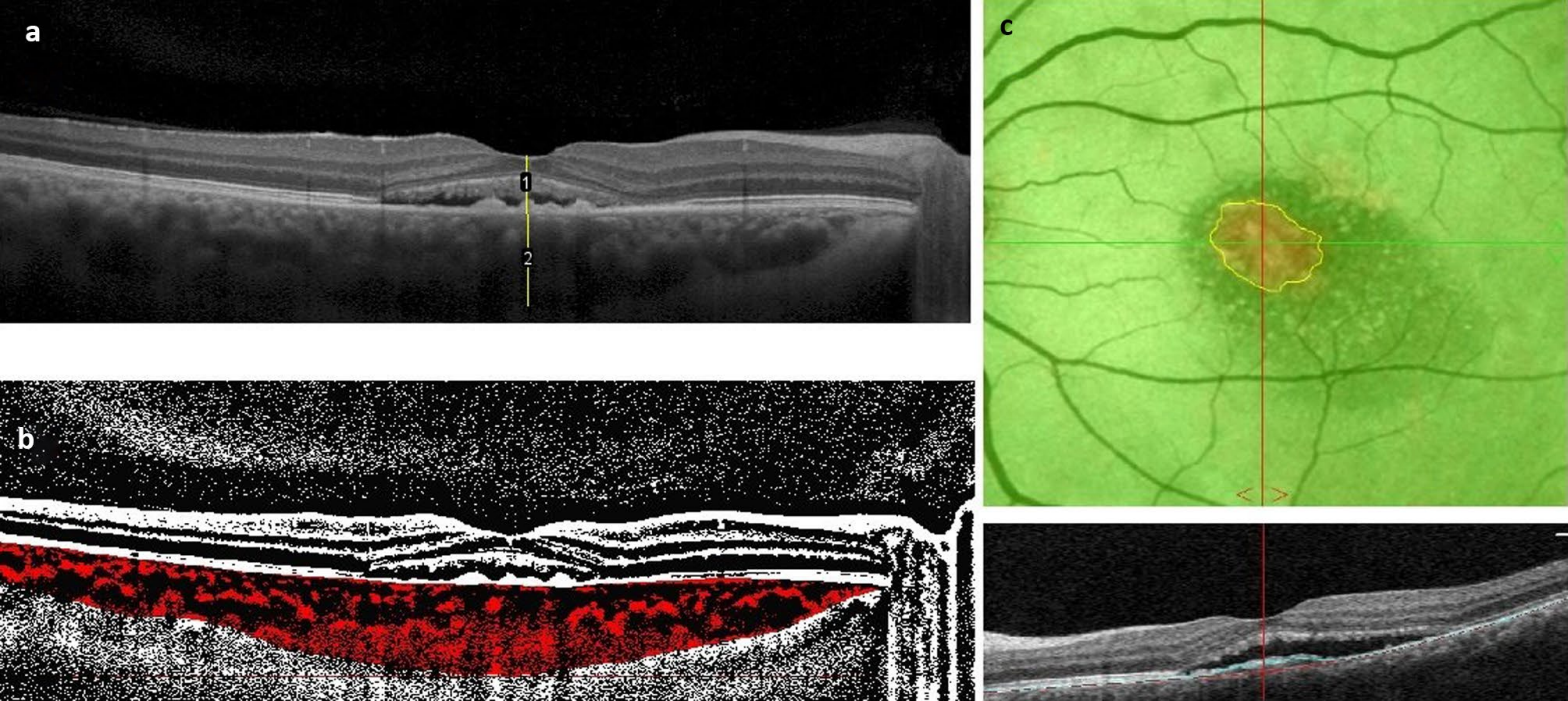
Measurement of retinal and choroidal thickness, choroidal vascular index (CVI) and pigment epithelial detachment (PED) area: Choroidal area is delineated followed by binarization of the region of interest (**a**). The image is then converted to RGB using color threshold. CVI is calculated by dividing areas without pixels as choroidal lumens to total choroidal area (**b**). En-face RPE elevation map, is generated by OCTA device. This map represents PED as hot regions. PED area is manually delineated, using FIJI software (**c**).

EDI-OCT was performed using Heidelberg spectralis OCT. All EDI OCT images were performed between 9 and 12 AM. Patients were positioned appropriately and a 5 × 5 mm image centered at the fovea was obtained for each eye. Similarly, OCTA images were obtained from the fovea (6 × 6 mm) area. Images with poor quality or from eyes with media opacity precluding acceptable image acquisition, were excluded from the study. Segmentation errors were manually corrected by a blind expert investigator (A.M). Image analysis was conducted by FIJI (an expanded version of ImageJ software, version 1.51 h; National Institutes of Health, Bethesda, Maryland, available at http://imagej.nih.gov/Fiji/). Both EDI-OCT and OCTA images were exported into FIJI software. Imaging measurements were performed by two investigators who were blind to the treatment label (K.F, A.M). The measurement of retina and choroid was performed manually in horizontal B-scan EDI-OCT images centered at fovea; thickness of the choroid was considered as the distance between the outer border of the RPE and the choroid–sclera border, and the thickness of retina was considered as the distance between internal limiting membrane and RPE (Fig. 1a).

In order to measure CVI, horizontal B-scan of EDI-OCT images centered at fovea were imported in FIJI were used. The borders of the choroid were selected using free hand tool of the software. The upper margin was RPE and the lower margin was the choroidoscleral border. The nasal margin was the temporal edge of the optic nerve head and the temporal margin was 8 mm from the temporal edge of the optic nerve head. To binarize choroidal area in OCT images, a modified Niblack method was used as previously described^12^. Briefly, three choroidal vessels with lumens larger than 100 $\mu{m}$ were randomly selected by the oval selection tool of the toolbar, and the average reflectivity of these areas was determined by the software. The average brightness was set as the minimum value to reduce the noise in the OCT image. The ROI was selected and set by the ROI manager in the OCT image. Then, the image was converted to 8 bits and adjusted by the auto local threshold of Niblack. The binarized image was reconverted to an RGB image, and the luminal area was determined using the color threshold tool. The light pixels were described as the choroidal stroma or interstitial area and the dark pixels were defined as the luminal area (LA). TCA, LA, and stromal area (SA) was automatically calculated. (Fig. 1b) Herein, we refer to the ratio of LA to TCA as the choroidal vascular index (CVI). They were calculated for all patients at baseline and month four following PDT.

Measurement of PED area was performed as previously described^13^. Briefly, En-face RPE elevation maps, obtained from OCTA imaging were used to depict the total PED area. Segmentation errors were manually corrected, considering the corresponding OCT B-scan. The images were imported to FIJI and scales were set accordingly. In these images, areas of RPE elevation appears as hot colors in heat map image. Consequently, borders of PED could be manually selected using the free hand tool of FIJI (Fig. 1c). A blind expert investigator (A.M) corrected the segmentation error and delineated the PED borders. PED area at baseline and follow-up images were measured and used for analysis.

### Statistical analysis

Data were entered into 'IBM SPSS Statistics for Windows, version 23 (IBM Corp., Armonk, N.Y., USA) and reported by descriptive statistics such as mean, standard deviation for quantitative values, and number and percentage for qualitative ones. The normality of the quantitative data was assessed using the Kolmogorov-Smirnov test. Inter- and intra-group analyses of normally-distributed data at baseline and four months after PDT were performed by independent t-test and paired t-test, respectively. Non-normally distributed data at baseline and four months after were compared by Mann-Whitney U test and Wilcoxon test. The analysis of covariance (ANCOVA) was used to adjust for baseline measures and to provide an unbiased estimate of the mean group difference of the 4 months results in two groups. Also, in this study, qualitative variables were compared using Fisher's exact test and Chi-square. A p-value less than 0.05 was determined as statistically significant. Statistical analysis was performed by a statistician blinded to group label (KF).

## Results

In the present study, we included 36 eyes; 24 eyes received ICGA-guided PDT and 12 eyes received FA-guided PDT. Twenty-eight eyes (77.8%) of males and 8 eyes (22.2%) of females were assessed. The mean age of patients was 44 ± 8.8 years. Table 1 demonstrates baseline characteristics of the patients. Vision, and retinal and choroidal parameters did not differ statistically between groups. however, spot sizes and numbers were significantly smaller and fewer in FA-guided compared to ICGA-guided group (2777 ± 1048 vs. 6016 ± 4503 µm, and 1.42 ± 0.90 vs. 2.50 ± 1.59; *P* value: 0.023, and 0.016; respectively). Hence, the total PDT energy was higher in ICGA-guided group (3283 ± 2520 J/cm2 vs. 5933 ± 3657 J/cm2, *p* value: 0.012).

**Table 1:** Baseline characteristics.

Variables	Overall N = 36	FA-based N = 12	ICG-based N = 24	*P* value
BCVA (logMAR)	0.49 ± 0.40	0.34 ± 0.26	0.56 ± 0.43	0.132^‡^
Age (years)	44 ± 8.8	44 ± 5	44 ± 10	0.865^†^
Gender (male)	28 (78%)	9 (75%)	19 (79%)	0.777*
CMT (µm)	334 ± 108	340 ± 115	332 ± 107	0.650^‡^
SFCT (µm)	354 ± 54	338 ± 38	363 ± 60	0.251^‡^
Choroidal Area (mm^2^)	0.47 ± 0.10	0.46 ± 0.13	0.48 ± 0.86	0.630^†^
CVI	81.16 ± 5.43	81.00 ± 4.44	81.25 ± 5.98	0.896^†^
PED area (mm^2^)	0.34 ± 0.49	0.19 ± 0.10	0.40 ± 0.58	0.959^‡^
PDT spot size (µm)	4939 ± 4002	2777 ± 1048	6016 ± 4503	**0.023**^**‡**^
PDT spot number	2.14 ± 1.47	1.42 ± 0.90	2.50 ± 1.59	**0.016**^**‡**^
PDT total energy (J/cm^2^)	5050 ± 3520	3283 ± 2520	5933 ± 3657	**0.012**^**‡**^

^†^independent sample t test; ^‡^Mann-Whitney U test; *chi square test.

*CMT* Central macular thickness, *SFCT* Subfoveal choroidal thickness, *CVI* Choroidal vascular index;

To assess the distribution of various patterns of FA leakage between our two study groups, we selected 28 eyes for whom both FA and ICG were available. Evaluating FA, 17 (47.2%) eyes had one discrete leaking area, 15 (41.7%) eyes had multiple discrete leaking areas, and 4 (11.1%) eyes showed diffuse leakage. Patterns of leakage in FA were equally distributed among FA-guided and ICGA-guided groups (p value: 0.990). Leakage areas in FA were compatible with ICG in 19 of 28 (68%) treated eyes. While six eyes showed a staining spot in FA of their asymptomatic fellow eyes, 21 of 28 (75%) eyes with ICGA had hyper-fluorescence in the asymptomatic fellow eyes. Hence, disparity between FA and ICG was observed in 9 of 28 (32%) treated eyes, whereas this was remarkable in 20 of 28 (72%) fellow eyes.

**Figure 2:**
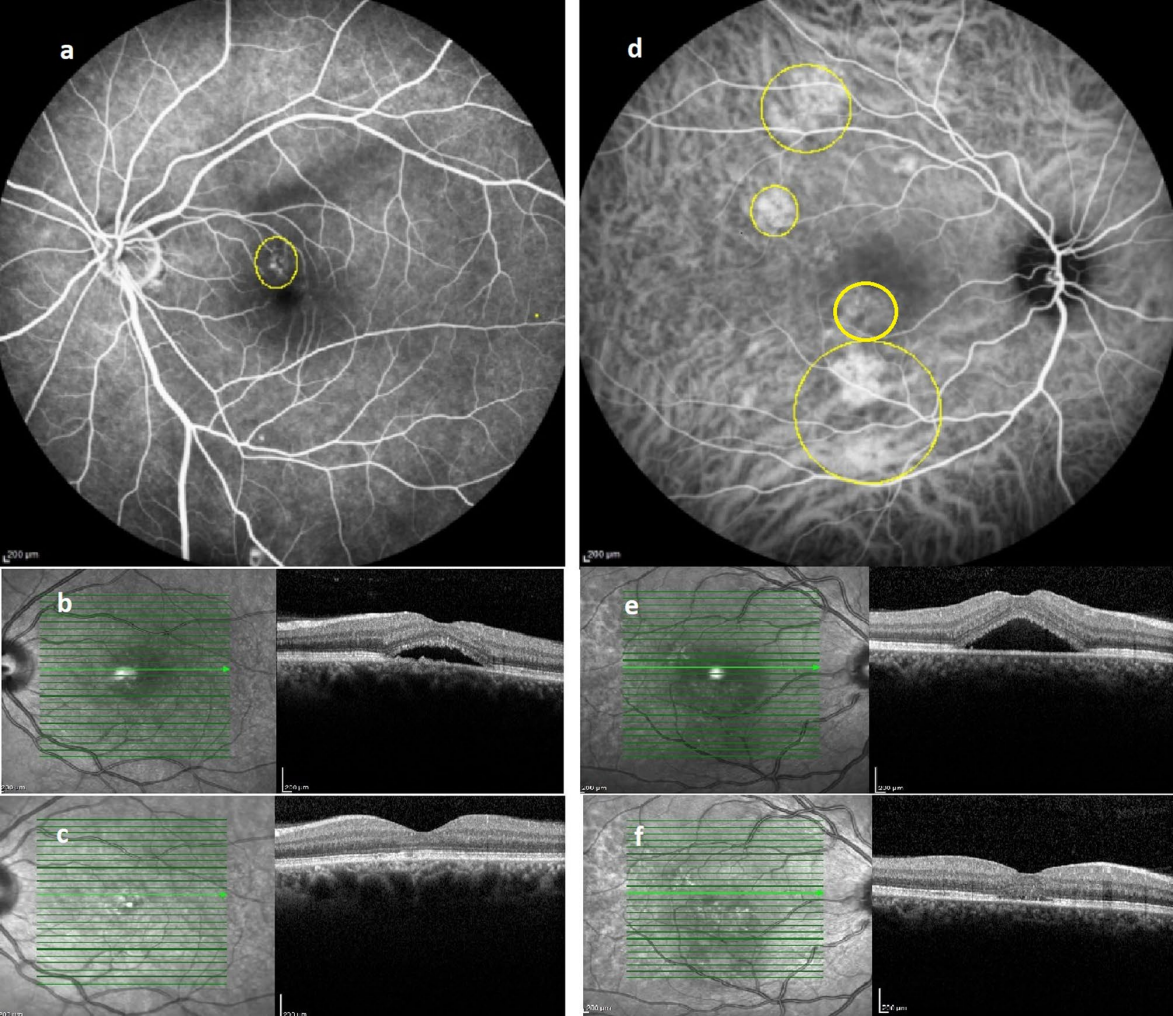
Representative image of patients in FA-based (left column) and ICGA-based (right column) PDT groups: FA; depicted area of leakage was treated with half dose PDT (**a**). Baseline OCT reveals foveal subretinal fluid (**b**), which completely resolved following treatment (**c**). ICGA; delineated areas corresponding to choroidal vascular hyperpermeability received half dose PDT (**d**). Baseline OCT reveals foveal subretinal fluid (**e**), which completely resolved following treatment (**f**).

Table 2 shows alterations in mean BCVA and imaging parameters at baseline and 4 months following treatment. We observed significant improvement in BCVA, as well as significant reduction in choroidal area, SFCT, CVI, and FSRF after PDT, in both FA-guided and ICGA-guided groups (Table 2). Intergroup analysis showed that the alterations of vision and imaging parameters do not significantly differ between the two groups. Figure 2 shows a representative image of our patients in each group.

**Table 2:** Alteration of vision and retinal and choroidal parameters following laser treatment.

Variables		Overall (mean ± SD)	FA–guided PDT (mean ± S)	IGCA–guided PDT (mean ± SD)	Intergroup *P*-value
BCVA (logMAR)	Baseline	0.49 ± 0.40	0.34 ± 0.26	0.56 ± 0.43	0.132^‡^
	4th month	0.33 ± 0.41	0.14 ± 0.13	0.43 ± 0.47	0.160^†^
	Change		− 0.19 ± 0.05	− 0.13 ± 0.02	
Intragroup *P*-value			0.005*	0.001*	
choroidal area (mm^2^)	Baseline	0.47 ± 0.10	0.46 ± 0.13	0.48 ± 0.86	0.630^§^
	4th month	0.39 ± 0.08	0.36 ± 0.08	0.40 ± 0.81	0.320^†^
	Change		0.09 ± 0.07	0.07 ± 0.05	
Intragroup *P*-value			0.001**	< 0.001**	
PED area (mm^2^)	Baseline	0.34 ± 0.49	0.19 ± 0.04	0.40 ± 0.16	0.959^‡^
	4th month	0.19 ± 0.39	0.07 ± 0.08	0.24 ± 0.46	0.930^†^
	Change		− 0.12 ± 0.03	− 0.16 ± 0.14	
Intragroup *P*-value			0.040*	0.002*	
SFCT (µm)	Baseline	354 ± 54	338 ± 38	363 ± 60	0.251^‡^
	4th month	275 ± 46	277 ± 44	274 ± 48	0.101^†^
	Change		61 ± 33	88 ± 51	
Intragroup *P*-value			0.001*	0.001*	
CVI	Baseline	81.16 ± 5.43	81.00 ± 4.44	81.25 ± 5.98	0.896^§^
	4th month	77.45 ± 5.04	76.16 ± 4.60	78.13 ± 5.23	0.174^†^
	Change		− 4.83 ± 2.83	− 3.12 ± 3.15	
Intragroup *P*-value			0.001**	0.001**	
CMT (µm)	Baseline	334 ± 108	340 ± 115	332 ± 108	0.650^‡^
	month	202 ± 63	195 ± 37	207 ± 74	0.478^†^
	Change		144 ± 109	124 ± 67	
Intragroup *P*-value			0.002*	0.001*	

Analyzed by: *Wilcoxon test, ^‡^ Mann–Whitney U, ^†^ANCOVA, ^§^Independent sample t test, **paired sample t test.

*SFCT* Subfoveal choroidal thickness, *CVI* Choroidal vascular index, *CMT* Central macular thickness.

With treatment, 34 (94.4%) eyes showed a reduction of FSRF; twenty-seven (75%) eyes had complete resolution of fluid. Two eyes, one in FA group and one in ICG group, with persistent FSRF, received another PDT treatament session; one eye had complete resolution of fluid and the other one showed a decrease in FSRF. Vision improvement was not statistically different among eyes with or without complete fluid resolution (− 0.15 ± 0.15 vs. − 0.16 ± 0.18 log MAR, respectively; *p* value: 0.827). Complete resolution of FSRF occurred similarly in both groups; 10 (83.3%) eyes in FA- and 17 (70.8%) in ICGA-guided groups, respectively (*p* value: 0.414). Any amount of fluid reduction was also similar between the two groups (*p* value: 0.607).

PED was present in 17 eyes, 5 in FA group and 12 in ICGA group. With treatment, 5 (29.4%) eyes become completely PED free, one from FA and 4 from ICGA group, and this was similar between the two groups (p value:0.582). None of the eyes experienced recurrence or exacerbation at the final visit.

## Discussion

We found a significant improvement in functional and anatomic parameters in both FA-guided and ICGA-guided PDT in our patients with non-resolving CSCR. However, the FA group achieved this with significantly lower laser energy required and smaller areas to be covered during PDT. We observed similar rates of BCVA improvement and fluid resolution in both groups. Additionally, we found that the reduction of CVI and PED area is the comparable between the two groups.

FA is a less expensive imaging modality than ICGA and is widely available in retina clinics. Based on our finding, FA can be employed for guiding PDT in patients with non-resolving CSCR, with smaller spot size and fewer number of laser spots compared to ICGA.

PDT is an effective treatment modality to tackle complications of non-resolving CSCR. However, it may cause ischemic damage to choriocapillaris with consequent RPE atrophy resulting in unfavorable visual outcome. The most effective and safe PDT protocol for CSCR treatment is still an ongoing challenge for ophthalmologists^14^. Several modifications involving time and fluence have been made to compensate for complications. However, occurrence of irreversible damage still remains a concern. Studies reported the possibility of choriocapillaris hypoperfusion and resultant decreased vision with PDT^15^. Bearing this in mind, the chances of complications would increase with the application of higher laser energy by including more extensive areas in PDT. According to Yannuzi et al.^7^ PDT area with spot sizes larger than 6000 µm, did not achieve visual improvement.

Due to intrinsic difference between ICGA and FA, subclinical cases of CSCR that have not developed RPE damage with subsequent leakage into SRF, may not show pathology in FA. In accordance with this fact that FA cannot penetrante to depict RPE/choroidal pathologies, we detected a higher rate of choroidal pathology using ICGA compared to FA in the asymptomatic fellow eyes. However, when the eye becomes symptomatic with FSRF, the disparity between FA and ICGA pathologic areas becomes less apparent (68% percent in asymptomatic fellow eyes versus 28% percent in treated eyes). The impact of this finding on the indication of treatment for asymptomatic eyes is still an ongoing debate. Although our short term results failed to reveal any benefit in treating the areas that show solely choroidal hyperpermeability and congestion.

The first attempt to incorporate FA in guiding PDT for eyes with chronic CSCR dates back to the investigation in 2020 by Koytak and colleagues, who reported vision improvement and reduction in retinal thickness at one year following FA-guided half dose PDT^16^. However, they did not have a control group of ICGA-guided treatment. Koytak et al., in a subsequent study, compared FA-guided versus ICGA-guided PDT in eyes with chronic CSCR, and showed vision improvement and reduction in retinal and choroidal thickness in both groups^9^. However, they possibly had a selection bias by recruiting the eyes with discrete leakage area in FA-guided group, and the more diffuse ones in ICGA-guided group. In our study, the baseline characteristics between the two groups, including the pattern of fluorescein leakage were not different among the two groups. Recently, Hayashida et al. compared FA-guided and ICGA-guided half-time PDT for the treatment of chronic CSCR and concluded that ICGA group give a more favorable anatomical outcome while the visual function was similar between their two groups. Their dissimilar result may be due to different treatment protocol. Recent studies assessed alternative imaging modalities for guiding focal laser photocoagulation for the treatment of CSCR. Maltsev et al. used OCT to find the area of RPE defect on which focal laser was applied. They showed both anatomical and functional success with this technique^17,18^.

To the best of our knowledge, this is the first study to report the comparison of alterations in CVI and PED area in FA-guided versus ICG-guided PDT in eyes with CSCR. Based on our finding, CVI and PED area reduced significantly following treatment. Based on our intergroup analysis, the decrease in CVI and PED area, was similar between the two groups. According to previous studies, eyes with acute and chronic CSCR have higher CVI compared to normal population^19,20^. Agrawal and colleagues observed a lower CVI in eyes with resolved CSCR compared to those with acute CSCR^19^. CVI accounts for a more reliable measurement for choroidal parameters as this ratio is least affected by diurnal variations of choroidal structure. This index has been shown to be plausible for monitoring disease activity^19^. In this regard, successful treatment of CSCR with half-dose PDT, results in vision improvement as well reduction of CVI and choroidal thickness^21^. Interestingly, undertreatment with half-dose-half-fluence PDT results in SFCT reduction, in the absence of vision improvement or CVI reduction^21^. In our study, vision improvement along with CVI reduction in both FA-guided and ICG-guided treated groups, indicates a sufficient initial treatment response.

The presence of PED is a common feature seen in eyes with CSCR, more prevalent in chronic forms of the disease^22,23^. There is a spatial relationship between the location of PED and the area of choroidal vascular abnormalities in eyes with CSCR^24^. Therefore, it is reasonable that following treatment of the abnormal choroid, a reduction in the PED area ensues. Our results demonstrated that 5 of 17 (29.4%) eyes with PED achieved a complete PED resolution, and 12 (70.6%) had a decreased PED area following treatment, which was similar between our two groups. This is consistent with previous studies that demonstrated PED improvement following PDT^25,26^.

Our study has some limitations. First of all, it is a non-randomized study, that may impose a bias to the results. Additionally unequal sample size between groups might be another source of bias. We applied the following approaches to compensate for these possible sources of bias. The patients had similar baseline characteristics in each group. We ensured that FA leakage patterns are not different among FA-guided and ICGA-guided groups. Additionally, the investigators who performed image and statistical analysis were blind to treatment label. A relatively small sample size and short follow-up time is another limitation of our study.

In conclusion, FA and ICGA-guided half-dose PDT showed similar functional and anatomical improvement in patients with non-resolving CSCR at 4 months following laser. FA guide is associated with applying less laser energy compared to ICGA guide and FA is more accessible and less expensive. The decision to surrogate FA with ICGA as a guide for PDT, still awaits further prospective validation with randomized controlled studies with longer follow up time.

## Data Availability

Derived data supporting the findings of this study including images, and statistical data set is available from the corresponding author on request.

## References

[1] Daruich A (2015). Central serous chorioretinopathy: Recent findings and new physiopathology hypothesis. Prog. Retin. Eye Res..

[2] Iacono P, Toto L, Costanzo E, Varano M, Parravano MC (2018). Pharmacotherapy of central serous chorioretinopathy: A review of the current treatments. Curr. Pharm. Des..

[3] Chan WM (2003). Choroidal vascular remodelling in central serous chorioretinopathy after indocyanine green guided photodynamic therapy with verteporfin: A novel treatment at the primary disease level. Br. J. Ophthalmol..

[4] Chan W-M, Lai TYY, Lai RYK, Liu DTL, Lam DSC (2008). Half-dose verteporfin photodynamic therapy for acute central serous chorioretinopathy: One-year results of a randomized controlled trial. Ophthalmology.

[5] Cardillo Piccolino F, Eandi CM, Ventre L, Rigault de la Longrais RC, Grignolo FM (2003). Photodynamic therapy for chronic central serous chorioretinopathy. Retina.

[6] Taban M, Boyer DS, Thomas EL, Taban M (2004). Chronic central serous chorioretinopathy: Photodynamic therapy. Am. J. Ophthalmol..

[7] Yannuzzi LA (2003). Indocyanine green angiography-guided photodynamic therapy for treatment of chronic central serous chorioretinopathy: A pilot study. Retina.

[8] Abouammoh MA (2015). Advances in the treatment of central serous chorioretinopathy. Saudi J. Ophthalmol. Off. J. Saudi Ophthalmol. Soc..

[9] Koytak A, Bayraktar H, Ozdemir H (2020). Fluorescein angiography as a primary guide for reduced-fluence photodynamic therapy for the treatment of chronic central serous chorioretinopathy. Int. Ophthalmol..

[10] Hayashida M, Miki A, Honda S, Nakamura M (2020). Comparison between the outcomes of fluorescein angiography-guided and indocyanine green angiography-guided half-time photodynamic therapy for central serous chorioretinopathy. Photodiagn. Photodyn. Ther..

[11] Ruiz-Del-Tiempo MP (2018). Anatomical retinal changes after photodynamic therapy in chronic central serous chorioretinopathy. J. Ophthalmol..

[12] Sonoda S (2014). Choroidal structure in normal eyes and after photodynamic therapy determined by binarization of optical coherence tomographic images. Invest. Ophthalmol. Vis. Sci..

[13] Ebrahimiadib N (2022). Flat irregular pigment epithelial detachment over time and outcome of different treatment regimens. Sci. Rep..

[14] Ladas ID (2018). Three-year results of fluorescein angiography-guided standard photodynamic therapy with multiple spots for central serous chorioretinopathy. Ophthalmol. Retina.

[15] Siaudvytyte L, Diliene V, Miniauskiene G, Balciuniene VJ (2012). Photodynamic therapy and central serous chorioretinopathy. Med. Hypothesis Discov. Innov. Ophthalmol. J..

[16] Koytak A (2010). Fluorescein angiography-guided photodynamic therapy with half-dose verteporfin for chronic central serous chorioretinopathy. Retina.

[17] Maltsev DS, Kulikov AN, Chhablani J (2019). Clinical application of fluorescein angiography-free navigated focal laser photocoagulation in central serous chorioretinopathy. Ophthalmic Surg. Lasers Imaging Retina.

[18] Maltsev DS, Kulikov AN, Chhablani J (2018). Topography-guided identification of leakage point in central serous chorioretinopathy: A base for fluorescein angiography-free focal laser photocoagulation. Br. J. Ophthalmol..

[19] Agrawal R (2016). Choroidal vascularity index in central serous chorioretinopathy. Retina.

[20] Degirmenci C, Akkin C, Yarimada S, Nalcaci S, Afrashi F (2020). Choroidal vascularity index in patients with chronic central serous chorioretinopathy. Retina-Vitreus.

[21] Park W, Kim M, Kim RY, Park YH (2019). Comparing effects of photodynamic therapy in central serous chorioretinopathy: Full-dose versus half-dose versus half-dose-half-fluence. Graefes Arch. Clin. Exp. Ophthalmol..

[22] Yang L, Jonas JB, Wei W (2013). Optical coherence tomography-assisted enhanced depth imaging of central serous chorioretinopathy. Invest. Ophthalmol. Vis. Sci..

[23] Mitarai K, Gomi F, Tano Y (2006). Three-dimensional optical coherence tomographic findings in central serous chorioretinopathy. Graefes Arch. Clin. Exp. Ophthalmol..

[24] Hirami Y (2007). Alterations of retinal pigment epithelium in central serous chorioretinopathy. Clin. Exp. Ophthalmol..

[25] Inoda S (2019). Half-dose photodynamic therapy for serous non-neovascular retinal pigment epithelial detachment. Clin. Ophthalmol..

[26] Roberts P (2016). Retinal pigment epithelial features in central serous chorioretinopathy identified by polarization-sensitive optical coherence tomography. Invest. Ophthalmol. Vis. Sci..

